# Randomized, Controlled Comparison of Advanced Hemostatic Pads in Hepatic Surgical Models

**DOI:** 10.1155/2014/930803

**Published:** 2014-03-04

**Authors:** Kevin M. Lewis, Jeff McKee, Alexandra Schiviz, Alexander Bauer, Martin Wolfsegger, Andreas Goppelt

**Affiliations:** ^1^Baxter Healthcare Corporation, One Baxter Parkway, Deerfield, IL 60015, USA; ^2^Baxter Innovations GmbH, Industriestraße 67, 1221 Vienna, Austria

## Abstract

Blood loss during hepatic surgery leads to poor patient outcomes. This study investigates the hemostatic efficacy of a novel sealing hemostatic pad (polyethylene glycol-coated collagen, PCC) and a fibrin sealant pad (fibrin-thrombin coated collagen, FTC) in a leporine hepatic segmentectomy and a porcine hepatic abrasion model. A segmentectomy was used to compare hemostatic success and hematoma incidence in 20 rabbits (10/group). Hepatic abrasions were used to compare hemostatic success up to 10 min after application in six pigs (42 lesions/group). In the segmentectomy model, PCC achieved 100% hemostatic success within 2 min (95% CI: 72.3% to 100%) and FTC achieved 80% hemostatic success within 3 min (49.0% to 94.3%). PCC had lower hematoma incidence at 15 min (0.0 versus 11.1%) and 24 h (20.0 versus 66.7%). In the abrasion model, PCC provided superior hemostatic success at 3 (odds ratio: 24.8, 95% CI: 8.86 to 69.2, *P* < 0.001), 5 (66.3, 28.5 to 153.9, *P* < 0.001), 7 (177.5, 64.4 to 489.1, *P* < 0.001), and 10 min (777.6, 148.2 to 4078, *P* < 0.001) leading to statistically significant less blood loss. The novel sealing hemostat provides faster and more sustained hemostasis than a fibrin sealant pad in a leporine hepatic segmentectomy and a porcine hepatic abrasion model of hepatic surgery.

## 1. Introduction

Patients undergoing hepatic resection can experience blood loss between 700 and 1,200 mL [1] leading to a 20–40% likelihood of receiving a transfusion [2]. These high blood loss and transfusion rates are associated with increased postoperative complications [3], increased rates of relaparotomy [4], prolonged hospital inpatient stay [3], increased likelihood of tumor recurrence [5, 6], decreased time to tumor reoccurrence [7], and greater likelihood of in-hospital mortality [3, 8]. Overall, blood loss is uniformly accepted as a predictor of patient outcome following hepatic resection, where lower blood loss has improved outcomes [4, 8, 9].

Bleeding in these patients is expected as the liver synthesizes and eliminates pro- and anticoagulation proteins [10, 11]. Acute and chronic hepatic disease leads to impaired synthesis of coagulation proteins (i.e., factors V, VII, and X–XII, fibrinogen, and prothrombin), thrombocytopenia, and excessive fibrinolysis [11, 12]. As a result, several different techniques are used during dissection and transection of hepatic parenchyma to minimize tissue perfusion and damage (e.g., Pringle maneuver, portal hypotension, surgical staplers, etc.). Inevitably, these methods have a transient or incomplete effect, which require treatment with a topical hemostatic agent [13, 14].

There are many options to treat the broad oozing surface of a hepatectomy, hepatic segmentectomy or hepatic sectionectomy [15], of which fibrin sealants have been preferred [16]. More recently, however, hemostatic pads are becoming the preference for open surgical procedures [17, 18]. The early adoption of hemostatic pads is limited in laparoscopic surgery due to unfavorable handling [18, 19]. As such, the need for versatile and effective hemostatic agents is well established [15] and continues to evolve given the economic demands on healthcare professionals and systems. Therefore, this study investigates the effectiveness of a new sealing hemostat in a nonheparinized leporine hepatic segmentectomy surgical model and a heparinized porcine hepatic abrasion surgical model.

The objective of this study was to compare the hemostatic effectiveness of a new polyethylene glycol-coated collagen pad (PCC) and a fibrinogen and thrombin-coated collagen pad (FTC) in two models of hepatic surgery. The alternative hypothesis tested was that PCC will have greater and more sustained hemostatic effectiveness than FTC.

## 2. Materials and Methods

### 2.1. Advanced Hemostatic Pads

**Figure 1:**
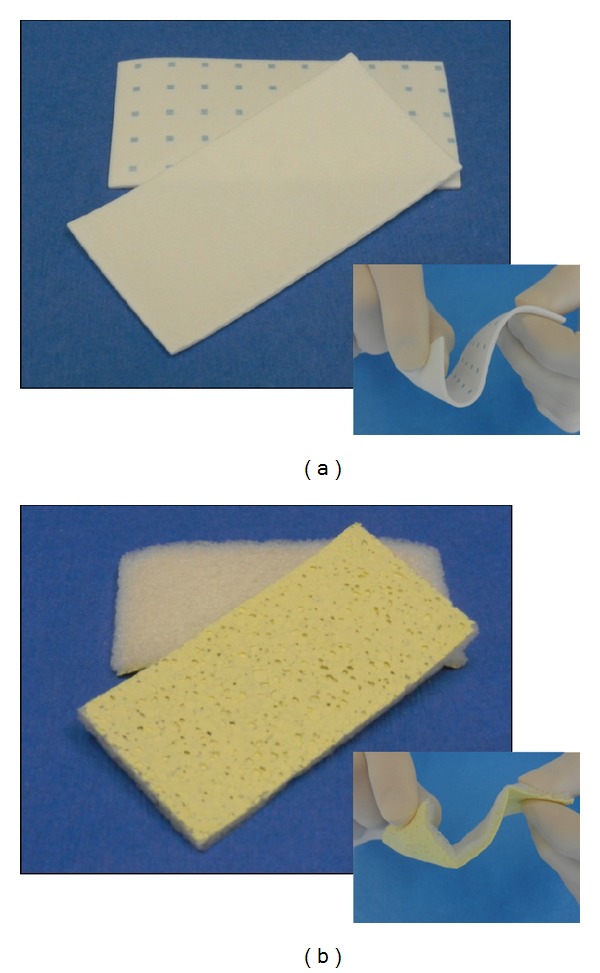
Hemopatch [sealing hemostat] is a pentaerythritol polyethylene glycol ether tetrasuccinimidyl glutarate (NHS-PEG) coated bovine collagen pad (a). TachoSil [absorbable fibrin sealant patch] is a human-derived fibrinogen and thrombin coated equine collagen pad (b). Insets demonstrate flexibility of each when manipulated.

The bovine collagen patch coated with a protein reactive pentaerythritol polyethylene glycol ether tera-succinimidyl glutarate (NHS-PEG) is Hemopatch (Baxter AG, Vienna, Austria) (PCC) (Figure 1(a)). When in contact with tissue, NHS-PEG forms covalent bonds between the collagen pad and tissue proteins, which seals the tissue and induces hemostasis in open and laparoscopic procedures. In both surgical models, PCC was approximated to tissue for 2 min.

The equine collagen pad coated with human-derived fibrinogen (5.5 mg/cm^2^) and thrombin (2.0 IU/cm^2^) is TachoSil (Takeda Pharmaceutical Company, Osaka-shi, OSK, Japan) (FTC) (Figure 1(b)). When wetted or in contact with tissue, thrombin cleaves fibrinogen forming a fibrin seal between the collagen pad and tissue surface. In both surgical models, FTC was approximated to tissue for 3 min according to the product insert [20].

### 2.2. Hepatic Surgical Models

Two hepatic surgical models were used to compare the hemostatic effectiveness of the hemostatic pads. A nonheparinized leporine hepatic segmentectomy surgical model mimicked the cut surface of a hepatectomy, sectionectomy, and segmentectomy, and a heparinized porcine hepatic abrasion surgical model mimicked a capsular tear during surgery. Both surgical models only used male animals to avoid coagulation variations due to estrus. All animal activities were performed according to the Guide for the Care and Use of Laboratory Animals and the United States Animal Welfare Act or Austrian Laws Governing Animal Experimentation in AAALAC accredited institutions following Animal Care and Use Committee approval.

The nonheparinized leporine hepatic segmentectomy model was performed by excising the distal tip of the medial hepatic lobe accessed through a celiotomy. The excision was performed using scissors without clamps or occlusion of the hepatoduodenal ligament. The cut surface was treated according to a randomization scheme not seen by the surgeon until the time of application. A single surgeon created and treated the lesions. A total of twenty male New Zealand white rabbits weighing 2.9 to 3.6 kg were used to create and treat 10 lesions/group. Each hemostatic pad was applied dry and approximated with gauze. The hemostatic pads extended the length of the cut surface and overlapped onto noninjured tissue by at least 1 cm. Hemostatic success was evaluated every 15 s after removing the gauze, every 30 s after 4.5 min, and then every 60 s after 7 min up to 15 min after application. Hemostatic success was predefined as no bleeding. If hemostatic success was not achieved within 15 min of application, the animal was removed from study. If hemostatic success was achieved within 15 min, the animal was recovered for 24 h. At 24 h, the lesion was assessed for rebleeding and presence of a hematoma and migration of the hemostatic pad.

The heparinized porcine hepatic abrasion model was performed by using a hand-drill (Robert Bosch Tool Corporation, Mt. Prospect, IL, USA) fixed with medium grade sandpaper (3 M, St. Paul, MN, USA) to create a series of two liver abrasions. Each 1 cm diameter, 3–4 mm deep abrasion was then treated according to a predefined, randomized scheme. Hemostatic pads (3 cm × 3 cm) were centered on the abrasion, applied dry, and approximated using gauze in pairs. A total of 42 abrasions/group were performed across six male pigs weighing between 31 and 38 kg. A single surgeon, who created and treated the abrasions, was blinded to the randomization scheme until time of application. At 3, 5, 7, and 10 min after application, the severity of bleeding was graded and the rate of blood loss was calculated in mL/min. Severity of bleeding was graded using an accepted grading scale [21, 22], where hemostatic success was predefined as no bleeding. Each animal was heparinized to 1.5–2x their baseline activated clotting time to mimic the coagulopathy secondary to hepatic disease [10–12].

### 2.3. Statistical Analysis

All analyses were performed with R version 2.15.3 [23]. The statistical significance was set to 5%.

#### 2.3.1. Nonheparinized Leporine Hepatic Segmentectomy Surgical Model

Hemostatic success and hematoma formation were measured in this preclinical model. Time to hemostasis was summarized using percentages of animals that experienced hemostasis within a specific time interval per group and displayed using Kaplan-Meier plots. Two-sided 95% confidence intervals (CIs) for the percentages of animals that experienced hemostasis within a specific time interval were also presented and calculated by the Wilson score method [24]. The incidence of hematoma at 15 min and 24 h after application is reported descriptively.

#### 2.3.2. Heparinized Porcine Hepatic Abrasion Surgical Model

Hemostatic success, difference in bleeding rate, and hematoma incidence were measured in this preclinical model. A generalized linear mixed model was used to analyze the probability of hemostatic success (i.e. bleeding scores of zero) after application over time, and a linear mixed model was used to analyze the bleeding rates in mL/min after application over time. Both models consisted of fixed effects covariates: (1) hemostatic pad, (2) time after application in minutes, (3) interaction of item × time to account for differences in hemostatic effectiveness over time between items, and (4) pretreatment bleeding rate in mL/min and an animal-specific intercept and an abrasion-specific intercept nested in animal as random effects. Additionally, the linear mixed model used for the bleeding rates consisted of an item-specific slope for modeling the time effect nested in abrasion and animal as random effect.

The generalized linear mixed model was performed using R function glmer [with option family = binomial] of R package lme4 [25]. Differences in hemostatic success between PCC and FTC within 3 to 10 min after application were assessed using model-estimated odds ratios, corresponding two-sided 95% Wald-type CIs, and two-sided *P*  values.

The linear mixed model was performed using R function lmer of R package lme4 [25]. Differences in bleeding rates between PCC and FTC within 3 to 10 min after application were assessed using model-estimated differences, corresponding two-sided 95% Wald-type CIs, and two-sided *P*  values. The two-sided *P*  values were calculated based on Markov Chain Monte Carlo (MCMC) simulations using R function pvals.fnc of R package languageR [26]. The incidence of hematoma is described descriptively.

## 3. Results

### 3.1. Nonheparinized Leporine Hepatic Segmentectomy Surgical Model

All excisions treated with PCC achieved hemostasis within 15 min of application. Nine of ten excisions treated with FTC achieved hemostasis within 15 min of application. The animal that did not achieve hemostasis was euthanized under anesthesia. The remaining nineteen animals, ten in the PCC group and nine in the FTC group, survived for 24 h.

**Figure 2:**
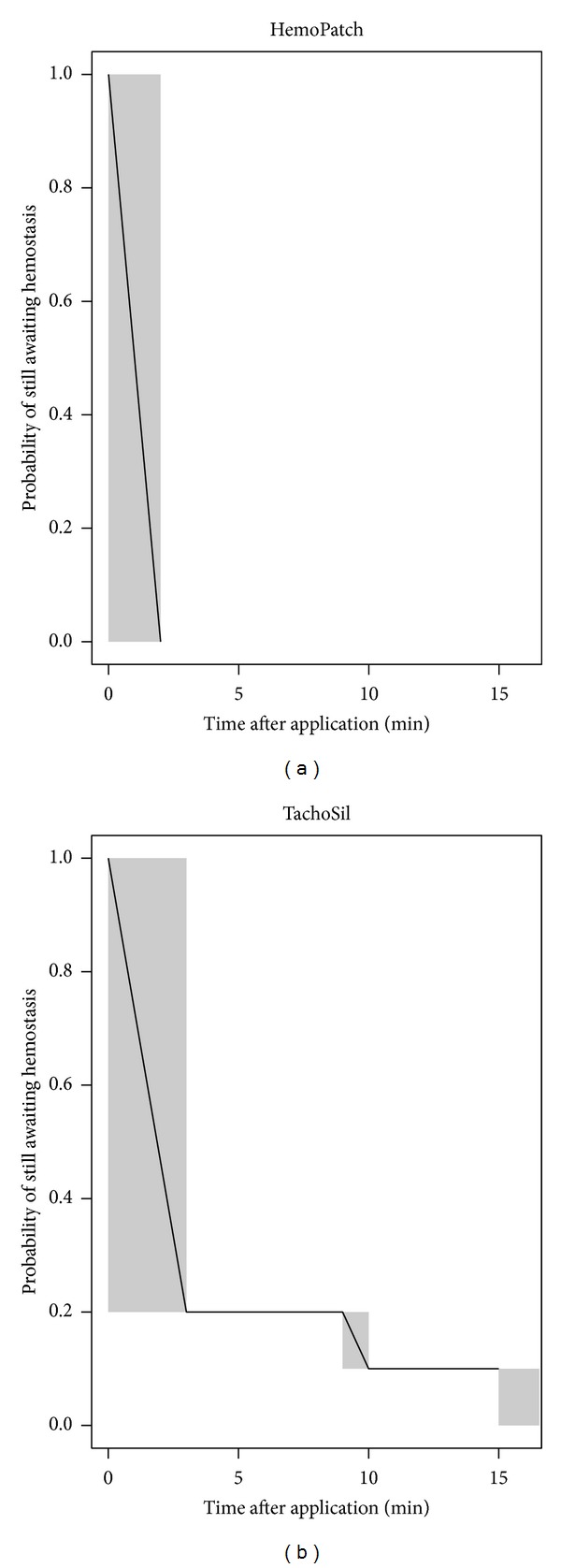
Kaplan-Meier plot for interval censored time of hemostasis of Hemopatch and TachoSil, where the time interval during which hemostasis occurred is shaded. Hemopatch, approximated for 2 min, achieved immediate hemostasis in 100% (95% CI: 72.3% to 100%) of applications; while TachoSil, approximated for 3 min, achieved immediate hemostasis in 80% (95% CI: 49.0% to 94.3%) of applications. One TachoSil-treated animal did not achieve hemostasis within the 15-minute observational period.

PCC-treated excisions had a greater percentage in achieving immediate hemostasis than FTC-treated excisions (100%, 95% CI: 72.3 to 100% within 2 min versus 80%, 49.0 to 94.3% within 3 min). At 15 min, PCC-treated excisions had 100% hemostasis, while FTC-treated excisions had 90% (Figure 2). Furthermore, PCC-treated animals had a 0% incidence of hematoma 15 min after application, whereas FTC-treated animals had 11.1%. The difference in hematoma increased at 24 h, where PCC-treated animals had a 20.0% incidence and FTC-treated animals had 66.7%.

### 3.2. Heparinized Porcine Hepatic Abrasion Surgical Model

**Figure 3:**
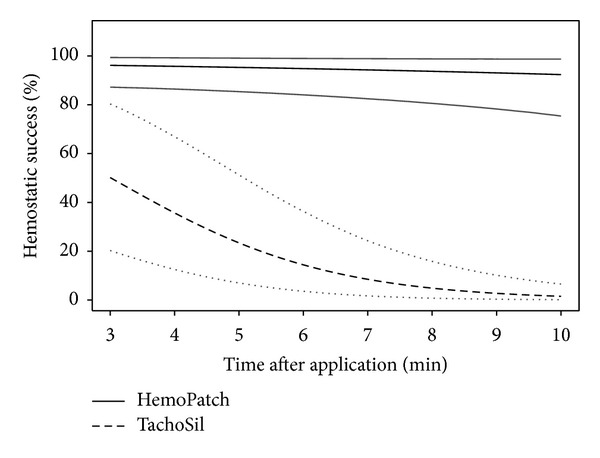
Calculated probability of hemostatic effectiveness (black) and corresponding bootstrap-type two-sided 95% confidence intervals (gray) for hemostatic success over time with Hemopatch (solid lines) and TachoSil (dashed lines) at the median bleeding rate.

**Figure 4:**
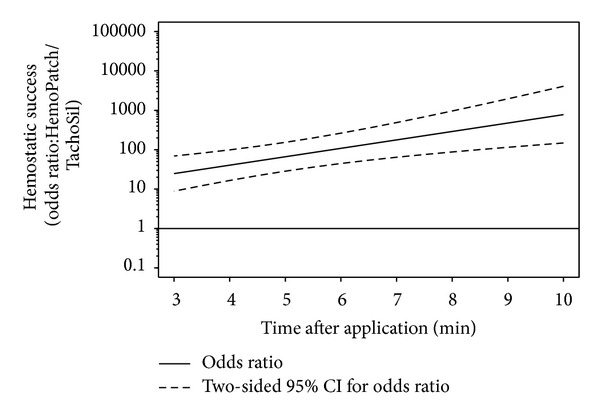
Odds ratio of hemostatic success and 95% confidence interval obtained from the generalized linear mixed effects model, where the odds ratio of hemostatic success of Hemopatch is divided by that of TachoSil. Hemopatch had a statistically significantly, at the 5% level, superior hemostatic success over time as the lower 95% confidence limit is greater than one.

**Figure 5:**
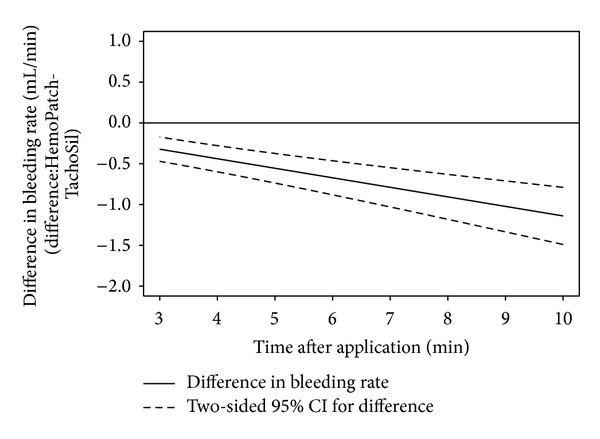
Difference in rate of blood loss from lesions treated with Hemopatch and those treated with TachoSil obtained from the linear mixed effects model, where the bleeding rate of TachoSil is subtracted from that of Hemopatch. Hemopatch had a statistically significant, at the 5% level, less bleeding rate over time as the upper 95% confidence limit is less than zero.

The calculated probabilities for hemostatic success for a median pretreatment bleeding rate of 2 mL/min were 96.1%, 95.3%, 94.3%, and 92.4% with PCC at 3, 5, 7, and 10 minutes after application. With FTC, these probabilities were 50.2%, 23.4%, 8.49%, and 1.53% (Figure 3). The odds ratio of hemostatic success indicates a statistically significant superiority of PCC to FTC at every time point after controlling the differences of pretreatment bleeding rates (Figure 4). PCC was 24.8 (95% CI: 8.86 to 69.2) times more likely to be effective at 3 min than FTC and 777.6 (95% CI: 148.2 to 4078) times more likely to be effective at 10 min (Table 1). Though not included in the study, the hemostatic performance of FTC improved after 10 min. The greater hemostatic success of PCC corresponded with a lower rate of blood loss, which was also statistically significant (Figure 5). PCC-treated abrasions had a bleeding rate of 0.32 mL/min (95% CI: −0.47 to −0.17) less than FTC at 3 min and 1.14 mL/min (95% CI: −1.49 to −0.79) less at 10 min (Table 1). PCC-treated abrasions also had a lower incidence of hematoma than FTC-treated abrasions (14.3 versus 42.9%).

## 4. Discussion

This study investigated hemostatic success, rate of blood loss, and hematoma incidence of hepatic lesions treated with hemostatic pads. PCC provided and maintained greater and faster hemostatic success with a lower rate of blood loss and incidence of hematoma than FTC. While the two surgical models are not absolutely predictive of clinical use, the models do allow a standardized comparison of hemostatic effectiveness.

In the hepatic segmentectomy model, the time to hemostasis was censored to not less than 2 min for PCC and not less than 3 min for FTC due to the different approximation times for each pad. The different approximation times mimicked the clinical use as described in the product insert and instructions for use. Despite the lower approximation time, PCC-treated lesions had a greater percentage of immediate hemostasis compared to FTC. The higher percentage of PCC is likely due to faster adherence to tissue. Since FTC relies on the conversion of fibrinogen to fibrin, additional time and approximation are likely required to achieve hemostasis. Hence, fibrin polymerization of FTC was slower and less consistent than the synthetic NHS-PEG polymerization of PCC in both models. The different adherence properties may also explain the differences in hematoma incidence.

In the hepatic abrasion model, the hemostatic success of FTC rapidly decreased over time from 50.2% at 3 min to 1.53% at 10 min after application due to its slower adherence, whereas hemostatic success of PCC was 96.1% at 3 min and 92.4% at 10 min after application. Furthermore, the lower hematoma incidence and blood loss with PCC demonstrate the advantage of its adherence properties. PCC rapidly forms covalent bonds between the collagen pad and injured tissue to stop bleeding independent of the patient's coagulation cascade, whereas FTC is dependent on the patient's ability to clot [27]. The effect of the coagulopathic state of the patient was observed in our models as well, wherein FTC performed better in the nonheparinized model. The faster adherence of PCC obviated ongoing blood loss and reduced hematoma formation that caused FTC to fail. The advantage of the novel adherence mechanism of PCC is demonstrated in both models. While these models replicated two clinical uses of the advanced hemostatic pads, they do not represent all bleeding scenarios. Therefore, additional studies are needed to compare these hemostatic agents in other surgical settings (e.g., cardiac, vascular, urological, etc.). In doing so, the utility of these agents in minimally invasive approaches should also be investigated.

Hepatic laparoscopic surgery is limited to small segmental resections due to fear of significant blood loss secondary to an inability for manual compression and of gas embolisms secondary to incomplete hemostasis of hepatic veins [20]. A sealing hemostat is likely able to address these challenges. PCC is pliable and supple, which can allow appropriate manipulations for use in endoscopic procedures. Once applied to tissue, PCC has a dual mechanism of action. The reactive NHS-PEG seals the collagen pad to tissue, which then activates platelets. The benefit of combining these properties—hemostasis and sealing—is extremely favorable for endoscopic surgery. Similar to PCC, FTC has a dual mechanism of action. The conversion of fibrinogen to fibrin by thrombin seals the collagen pad to tissue and the collagen pad stimulates platelet activation. However, the sealing affect requires greater time and the collagen construct of FTC lacks the pliability and suppleness for efficient endoscopic use.

The liver is extremely vascularized due to its sinusoidal structure, which lacks smooth muscle capable of vasoconstriction [28]. Therefore, hepatic resections, trauma, and lacerations create wide, raw surfaces with multiple bleeds that cannot be sutured or ligated. Furthermore, liver disease has the sequelae of coagulopathy due to reduced capability of synthesizing procoagulant proteins [10–12]. The strength of our surgical models is that they replicated these conditions. The leporine hepatectomy model replicated the wide, raw surfaces of bleeding, and the porcine hepatic abrasion model replicated the tissue damage and coagulopathy secondary to liver disease. While both PCC and FTC have hemostasis and sealing abilities, PCC provided greater hemostasis faster than FTC with a lower blood loss and incidence of hematoma in both models.

The beneficial effects of topical hemostatic agents are conferred in the scientific literature, which suggest that these agents reduce time to hemostasis and lower rates of perioperative RBC transfusions [29]. These findings directly result in cost savings for payers and healthcare systems. A 2005 publication estimated the cost of operating time to be 51€/min ($66/min) [30] and a 2008 publication estimated the cost of a RBC transfusion to be 1069€/patient ($1388/patient) [31]. The reduced time to hemostasis and the greater hemostatic success of PCC are then likely to have added benefit for healthcare systems. Further, the improved effectiveness of PCC relative to FTC benefits patients undergoing hepatic resections, as lower intraoperative blood loss, is associated with improved outcomes [4, 8, 9].

Though these hemostatic pads have not been compared in human patients, this data indicates that the novel sealing hemostat (PCC) provides faster and more sustained hemostasis, less blood loss, and lower hematoma formation than a fibrin sealant pad (FTC). The difference in efficacy is due to the rapid sealing and hemostatic action of PCC, which establishes it as a next generation advanced hemostatic pad.

## Figures and Tables

**Table 1:** Differences between Hemopatch and TachoSil at 3, 5, 7, and 10 minutes after application in hemostatic success and bleeding rates after controlling for pretreatment bleeding rate. Hemopatch had superior hemostatic success relative to TachoSil at all-time points based on the odds ratio of success, where a lower 95% confidence interval greater than one indicates superiority of Hemopatch. Hemopatch had significantly less blood loss than TachoSil at all-time points based on the difference of blood loss, where an upper 95% confidence interval less than zero indicates statistical significance.

Time after application	Hemostatic success (odds ratio: Hemopatch/TachoSil)			Difference of bleeding rate (difference: Hemopatch-TachoSil)		
	Odds ratio	Two-sided 95% confidence interval for odds ratio	Two-sided *P* value	Difference (mL/min)	Two-sided 95% confidence interval for difference (mL/min)	Two-sided *P* value
3	24.8	8.86 to 69.2	<0.001	−0.32	−0.47 to −0.17	<0.001
5	66.3	28.5 to 153.9	<0.001	−0.56	−0.74 to −0.37	<0.001
7	177.5	64.4 to 489.1	<0.001	−0.79	−1.03 to −0.55	<0.001
10	777.6	148.2 to 4078	<0.001	−1.14	−1.49 to −0.79	<0.001
